# Caregiving Arrangements and Health Outcomes of Chinese Older Adults With Disability in Cross-National Settings

**DOI:** 10.1093/geroni/igaa057.2702

**Published:** 2020-12-16

**Authors:** Jing Wang, Bei Wu

## Abstract

This symposium focuses on the wellbeing of older adults with disability/cognitive impairment and their family caregivers. More specifically, it aims to understand how familly support, community resources utilization, internal migration, and immigrant status impact older adults’ caregiving arrangement, health outcomes and end-of-life preferences and family caregivers’ caregiving burden in China and the U.S. The first study explored how perceived spousal relationships and support impact dyadic experiences of living with cognitive impairment through a person-centered care lens during a three-year period. The second presentation examined the association between adult children’s support and the trajectories of depressive symptom level among Chinese older adults with disabilities. The third investigated how family relationship and immigrant status matter in advanced care planning (ACP) engagement and end-of-life preferences over burial plan among US-born and foreign-born older Chinese Americans living in Honolulu, Hawaii. The fourth study study explored family caregivers’ caregiving burden for community-dwelling patients with dementia and its associated factors. The last study conducted an inventory of longitudinal aging survey datasets to stimulate research on intersection of migration and caregiving arrangement. It paved the way to use existing high-quality datasets to examine the significant impact of massive rural-to-urban migration on caregiving arrangement among Chinese older adults. This symposium presents empirical evidence of the impact of family, migration and culture-related factors on caregiving arrangement and health outcomes of Chinese older adults. The presenters emphasize the importance of providing family-centered care and design culturally sensitive interventions to improve the health outcomes of older adults.

